# Efficacy and safety of radical cystectomy with ileal conduit for muscle-invasive bladder cancer in the elderly: a multicenter retrospective study

**DOI:** 10.3389/fonc.2024.1402360

**Published:** 2024-07-23

**Authors:** Heqian Zhang, Anrui Li, Wentao Wang, Songlin Xu, Changfu Li, Lichen Teng

**Affiliations:** Department of Urology, Harbin Medical University Cancer Hospital, Harbin, China

**Keywords:** bladder cancer, urinary diversion, radical cystectomy, ileal conduit, elderly patients, security, effectiveness, transfer

## Abstract

**Objective:**

Radical cystectomy with ileal conduit is the current mainstay of treatment for muscle-invasive bladder cancer and is also a high-risk procedure. Existing studies have limited targeted assessment of the efficacy and safety of this procedure, and the patient population appropriate for this procedure is still poorly defined. We sought to longitudinally analyze differences in the efficacy and safety of radical cystectomy with ileal conduit by age subgroups to assess whether the age factor should be used as an exclusion criterion when selecting this procedure.

**Materials and methods:**

We retrospectively examined the clinicopathological data of patients with MIBC treated with RC with IC at the Cancer Hospital of Harbin Medical University between February 2014 and October 2023. Additionally, we utilized clinical and pathological data from the SEER database (2000-2020) for external validation of our findings. Patients were categorized into elderly (≥70 years at diagnosis) and non-elderly (<70 years) groups. Statistical analyses included t-tests, non-parametric tests for continuous data, chi-square tests for categorical data, and Kaplan-Meier survival analysis.

**Results:**

In this study, 152 patients were included: 119 were categorized as non-elderly and 33 as elderly. For external validation, data from 416 patients in the SEER database were analyzed, with 172 classified as non-elderly and 244 as elderly. The results indicated that elderly patients were more likely to require ICU transfer postoperatively but exhibited a lower incidence of stoma inflammation. Additionally, both the data from our center and the external validation from the SEER database showed a concordance in cancer-specific survival (CSS) between the elderly and non-elderly groups. The efficacy of RC with IC was comparable in both elderly and non-elderly patients.

**Conclusion:**

For longitudinal age subgroups, RC with IC for both elderly and non-elderly MIBC had good efficacy and safety, and good quality of life after surgery. Although there are surgical and perioperative risks in elderly patients, there is no significant difference compared with non-elderly patients. In elderly patients requiring RC for bladder cancer, IC should remain the preferred mode of urinary diversion, and old age should not be used as an absolute exclusion criterion for IC.

## 1 Introduction

Bladder cancer is the ninth most common cancer globally, with approximately 610,000 new cases and 220,000 deaths annually, as reported in the 2022 Global Cancer Statistics (1). The disease predominantly affects the elderly; statistical data reveal that the median age at diagnosis is 73 years, and individuals over 65 years old constitute about 75% of all bladder cancer patients. As bladder cancer incidence increases with age, and with the global population aging, it is projected that the number of elderly patients will continue to rise. (2).

Muscle-invasive bladder cancer(MIBC) represents a severe pathological stage of bladder cancer. Radical cystectomy(RC) with pelvic lymph node dissection is the established standard treatment for this condition. After RC, the choice of urinary diversion method is crucial and must be tailored to each patient’s specific circumstances. The primary methods of urinary diversion include cutaneous ureterostomy, ileal conduit(IC), and neobladder construction. Among these, the IC is the most widely adopted globally due to its favorable impact on urinary function and quality of life, as well as its relatively low demands on patient compliance (3).

Previous studies on the use of RC for treating MIBC in elderly patients have shown conflicting results. Some research indicates that older patients experience higher complication rates (4), and that increasing age correlates with poorer survival outcomes (2, 5). However, other studies assert that RC is safe for elderly patients, arguing that it does not necessitate routine intensive postoperative monitoring and is associated with acceptable perioperative morbidity and mortality rates (6). Given the conflicting results from previous studies and the lack of focused analysis on specific urinary diversion modalities, we conducted a retrospective and follow-up study of both elderly and non-elderly patients who underwent RC with IC at our institution. Additionally, we utilized data from the SEER database to externally validate our findings.

## 2 Materials and methods

### 2.1 Data from our center

#### 2.1.1 Patient choice

Inclusion criteria were patients with bladder cancer who underwent RC with extracorporeal IC from February 2014 to October 2023 at the Affiliated Cancer Hospital of Harbin Medical University and defined patients whose age was greater than or equal to 70 years old at the time of diagnosis as elderly patients and patients younger than 70 years old as non-elderly patients. Exclusion criteria: ① patients with bladder cancer who had undergone neoadjuvant chemotherapy or neoadjuvant immunotherapy before surgery, and patients with recurrent bladder cancer who were still in the cycle of cystoperfusion chemotherapy ② patients with non-muscle invasive bladder cancer with postoperative pathological confirmation of pT stage less than T2 (Due to the high risk of progression to pT2, pT is still included in this study) ③ patients with distant metastases confirmed by imaging before surgery, i.e., clinical stage Patients with bladder cancer with cM>0 ④ Patients who had been lost to telephone follow-up and those who were unable to complete the questionnaire by answering the questions on their own or without the assistance of a relative to complete the questionnaire.

#### 2.1.2 Surgical procedures

Surgeries were performed according to Chinese urological guidelines (7), The general procedure of laparoscopic RC with IC is to clear the pelvic lymph nodes → resection the bladder → establish the ileocecal channel, and open IC of the bladder with IC to resect the bladder → clear the pelvic lymph nodes → establishment of the ileocecal channel. The surgical scope of both procedures is the bladder and surrounding tissues, the distal ureter, and pelvic lymph on both sides, including the prostate and seminal vesicles in male patients; and the uterus, part of the anterior vaginal wall, and adnexa in female patients. Intraoperative rapid frozen pathology was performed for any suspected positive tissue breaks. Pelvic lymph nodes were cleared medially to the ureter, laterally to the genitofemoral nerve, proximally to the bifurcation of the common iliac vessels, and distally to the deep spinothalamic vein. For the IC, a 15 cm length of ileal intestinal segment 15 cm from the ileocecal region was selected, preserving the mesenteric blood supply, with a distal stoma in the right rectus abdominis muscle paraspinalis, and proximally connecting to the right and left ureters of the lateral anastomosis. Intraoperative drains were placed, one abdominal drain, one pelvic drain, and one transurethral pelvic drain.

#### 2.1.3 Collection and follow-up of clinical data

Patient information, including clinical, pathological, and surgical characteristics, is extracted from the electronic medical record (EMR) system.

Post-operative follow-up was done by telephone. If the patient died, the cause and time of death were asked, and if the death was related to the bladder tumor, the patient was further asked about the time of diagnostic recurrence; if the patient survived, the patient was investigated for postoperative disease up to the last week, with questions about complications and the patient’s tumor progression. Complications included urinary tract infections, gastrointestinal dysfunction, and ileal access fistulae within 1 week of the follow-up time. Urinary tract infection was defined as patients presenting with fever, chills, low back pain, and a bacterial colony count ≥10^5 CFU/ml confirmed by urine bacteriological culture. Gastrointestinal dysfunction was defined as patients presenting with abdominal pain, abdominal cramps, vomiting, and cessation of defecation with imaging to rule out other acute abdominal conditions. Stoma injury was categorized as complications affecting both the stoma and its adjacent skin. Specifically, stoma skin inflammation was identified as irritant contact dermatitis, clinically presenting with symptoms such as redness, swelling, hard nodules, papules, and herpetic lesions on the stoma and surrounding skin, as reported by patients within one week of follow-up. Stoma obstruction was defined by imaging-confirmed fluid accumulation in unilateral or bilateral kidneys or ureters, caused by stones in the stoma or other factors. Additionally, stoma hemorrhage was described as bleeding from the stoma due to causes other than stones, infections, or inflammation, including bleeding related to stoma care operations or unexplained gross hematuria. The primary endpoints for survival analysis were overall survival (OS), recurrence-free survival time (RFS), and cancer-specific survival (CSS). OS is the time between the initial MIBC diagnosis and the time of death from any cause or the time of the last follow-up visit. RFS is the time between the initial MIBC diagnosis and the time of clinically confirmed recurrence is the time between the initial MIBC diagnosis and death due to MIBC.

Patients were also asked to complete the modified Complications Clavien Classification System (CCS) at 30 days postoperatively and 90 days postoperatively at follow-up and to complete the FACT-BL questionnaire asking about their recent quality of life. The Clavien Classification System is a strategy proposed by Clavien et al. in 1992 to rank surgical complications based on the consequences of treatment (8), it has been popularized and widely used for postoperative assessment in urology, especially after modification and improvement in large cohort studies (9, 10). The FACT-BL is a bladder cancer-specific quality of life (QOL) measure that includes five dimensions: physical health, social/family health, emotional health, functional health, and bladder cancer-specific concerns, with specific questions scored on a 5-point scale (11), with higher scores being associated with better quality of life. The subscales were summed to give a total FACT-BL score (range 0 - 156) after the patient completed the questionnaire to assess the patient’s quality of life in the last 1 week (12).

### 2.2 Seer database data

Patients diagnosed with bladder cancer between 2000 and 2020 were identified by the Surveillance, Epidemiology, and End Results (SEER) Cancer Registry using the SEER*STAT (version 8.4.3) software, following approval from the National Cancer Institute. Patients were identified using the original site of the tumor as the bladder. The patient population was selected to be essentially the same as the patient population in our center, and the inclusion criteria were (1) pathological diagnosis of bladder cancer from 2000-2019; (2) complete clinical information; and (3) patients of patients who underwent RC with IC. The exclusion criteria were as follows: (1) pT stage less than 2 and pM stage greater than 0; (2) missing or unknown clinical information; and (3) patients who had undergone preoperative radiotherapy and neoadjuvant chemotherapy. The following clinicopathological factors were retrieved as variables for analyzing differences between groups: age, sex, TNM stage, survival time, and cause of death. Since the seer database does not contain detailed perioperative laboratory tests and the recovery process of the patients, only external validation of efficacy, i.e., OS and CSS, was performed.

### 2.3 Statistical analysis

Descriptive statistics were used to describe demographic and clinical characteristics. For measurements that conformed to a normal distribution with chi-square, differences between groups were analyzed using the independent samples t-test, otherwise, the Mann-Whitney U non-parametric test was used. For count data, the chi-square test was used to analyze differences between groups. For outcomes that included time of occurrence the Kaplan-Meier method was used to plot survival curves and analyze differences between groups. All the above analyses were done using SPSS (version 20, IBM, Armonk, NY, United States). A P<0.05 was considered by statistical significance.

## 3 Results

### 3.1 Clinical baseline characteristics of patients

All 152 patients included in our center underwent RC of the bladder with an IC at the Cancer Hospital of Harbin Medical University and were followed up. Most of the patients were male (n=131,86%). Patients were divided into elderly patients (n=33,22%) and non-elderly patients (n=119,78%) according to whether they were >70 years old at the time of diagnosis of bladder cancer. There was no statistically significant difference between the two groups in terms of pathological stage and whether it was accompanied by poor histological differentiation (P=0.618, P=0.524). The basic information and clinical characteristics of the patients are shown in **Table 1**.

**Table 1:** Clinical, pathological, and surgical types of included patients.

		Entire cohort (n=152)	<70 (n=119)	≥70 (n=33)	Pvalue
Age		63 gel.3	62 2el.2	75.7 5.7.5	<0.001
Male		131 (86)	106 (89)	25 (75)	0.94
BMI		23.8 (20.8-26.7)	24 (21.2-26.8)	22.7 (20-26.8)	0.392
Symptomatic					
	hematuria	117 (76)	89 (74)	28 (84)	0.225
	irritable bladder sign	102 (67)	81 (68)	21 (63)	0.632
Smoking history		68 (44)	59 (49)	9 (27)	**0.023**
Addicted to alcohol		20 (13)	16 (13)	4 (12)	1
Histological					0.618
	undesirable divergence	15 (9)	13 (10)	2 (6)	
	squamous	7 (4)	5 (5)	2 (6)	
	adenoid	6 (4)	6 (4)	0	
	sarcomatoid	1 (0.6)	1 (0.8)	0	
	neuroendocrine cell	1 (1)	1 (1)	0	
Patdologic T stage					0.524
	2a	77 (51)	60 (50)	17 (52)	
	2b	19 (13)	13 (11)	6 (18)	
	3a	32 (21)	25 (21)	7 (21)	
	3b	9 (6)	8 (7)	1 (3)	
	4a	12 (8)	10 (8)	2 (6)	
	Tis	3 (1)	3 (3)	0	
Patdologic N stage					0.948
	N0	129 (85)	101 (85)	28 (85)	
	N1	8 (5)	7 (6)	1 (3)	
	N2	15 (10)	11 (9)	4 (11)	
Surgical approach					0.023
	Laparoscopy	99 (65)	72 (61)	27 (82)	
	Open	53 (36)	47 (39)	6 (18)	

Age is expressed as mean ± standard deviation, BMI as median (interquartile range), and all others as n (%).

The bold values means that they are statistically significant.

### 3.2 Preoperative assessment of patients, surgical characteristics, perioperative risks, and postoperative complications

In this study, we initially compared the preoperative risk scores between two patient groups. The results demonstrated statistically significant differences in ASA, CCI, and PNI scores (ASA: P=0.001, CCI: P=0.017, PNI: P<0.001). Specifically, elderly patients had higher CCI scores compared to the non-elderly group (CCI 1 [12% vs. 19%], CCI 2 [18% vs. 9%], CCI ≥3 [15% vs. 1%]; P=0.017), higher ASA scores (ASA 1 [42% vs. 70%], ASA 2 [24% vs. 23%], ASA 3 [21% vs. 6%], ASA 4 [12% vs. 1%]; P=0.001), and lower PNI scores (43.5 vs. 47.9; P<0.001). These findings are detailed in **Table 2**.

**Table 2:** Patient pre-surgical risk assessment.

		Entire cohort (n=152)	<70 (n=119)	≥70 (n=119	Pvalue
CCI					**0.017**
	0	102 (67)	84 (71)	18 (55)	
	1	27 (18)	23 (19)	4 (12)	
	2	17 (11)	11 (9)	6 (18)	
	3	6 (4)	1 (1)	5 (15)	
ASA					**0.001**
	1	97 (64)	83 (70)	14 (42)	
	2	35 (23)	27 (23)	8 (24)	
	3	15 (10)	8 (6)	7 (21)	
	4	5 (3)	1 (1)	4 (12)	
NNIS					0.12
	0	2 (1)	1 (1)	1 (3)	
	1	12 (8)	11 (9)	1 (3)	
	2	84 (55)	68 (57)	16 (48)	
	3	26 (17)	20 (17)	6 (18)	
	4	18 (12)	13 (11)	5 (15)	
	5	10 (7)	6 (5)	4 (12)	
PNI		46.7 (43.9-50.6)	47.7 (44.9-51.5)	43.2 (40.0-46.8)	**<0.001**

PNI is expressed as median (interquartile range), and CCI score, ASA score, and NNIS score are expressed as number of patients (percentage).

The bold values means that they are statistically significant.

The analysis of patients’ surgical characteristics revealed that the only statistically significant difference between the groups was the likelihood of ICU transfer post-surgery, with elderly patients being at a higher risk ([30% vs. 6%]; p<0.001). There were no significant differences in surgery duration (370 vs. 335 minutes; p=0.459), intra-operative hemorrhage (300 ml vs. 200 ml; p=0.071), or the rate of blood transfusions (33% vs 42%; p=0.368), as detailed in **Table 3**. Subgroup analyses were conducted to assess potential biases due to surgical modality and operator factors. The results indicated that variations in surgical modality and operator did not significantly affect the risk of postoperative ICU transfers, as shown in **Supplementary Table 1**. Differences in reasons for ICU transfers and the duration of ICU stays are presented in **Supplementary Table 2**.

**Table 3:** Surgical safety - surgical features and perioperative risks.

		Entire cohort (n=152)	<70 (n=119)	≥70 (n=33)	Pvalue
Surgical features					
	duration of surgery (min)	355 (315-415)	370 (330-420)	335 (280-358)	0.459
	haemorrhage (ml)	300 (200-500)	300 (200-500)	200 (200-400)	0.071
	blood transfusion	61 (40)	50 (42)	11 (33)	0.368
	Transferred to ICU	17 (11)	7 (6)	10 (30)	**<0.001**
Parenteral nutrition (days)		6 (5-8)	6 (5-8)	6 (4-8)	0.559
Antibiotics (days)		7 (6-8)	7 (6-8)	7 (6-7)	0.426
Drainage					
	abdominal drainage (days)	7 (5-9)	7 (5-9)	7 (4.5-8)	0.248
	pelvic drainage (days)	7 (6-9)	8 (6-9)	7 (5-8)	0.064
	urethral drainage (days)	9 (8-11)	9 (8-11)	9 (8-10)	0.93
Length of hospitalization		16 (13-20)	16 (13-21)	15 (11.5-17.5)	0.058
Post-operative length of hospitalization		10 (8-14)	10 (8-14)	10 (8-14)	0.113
cost (￥)		6.7 (6.1-7.5)	6.6 (6.1-7.5)	6.9 (6.2-7.9)	0.719

Measures are expressed as median (interquartile range) and counts are expressed as number (percentage).

The bold values means that they are statistically significant.

For a comparison of perioperative risks between elderly and non-elderly patients, the length of parenteral nutrition (6 days vs. 6 days; p=0.559), antibiotic use (7 days vs. 7 days; p=0.426), and the duration of drain drainage (abdominal drainage [7 days vs. 7 days; p=0.248], pelvic drainage [8 days vs. 7.9 days; p=0.064], urethral drainage [9 days vs. 9 days; p=0.93]) showed no statistically significant differences between the two groups. These findings are detailed in **Table 3**.

**Figure 1:**
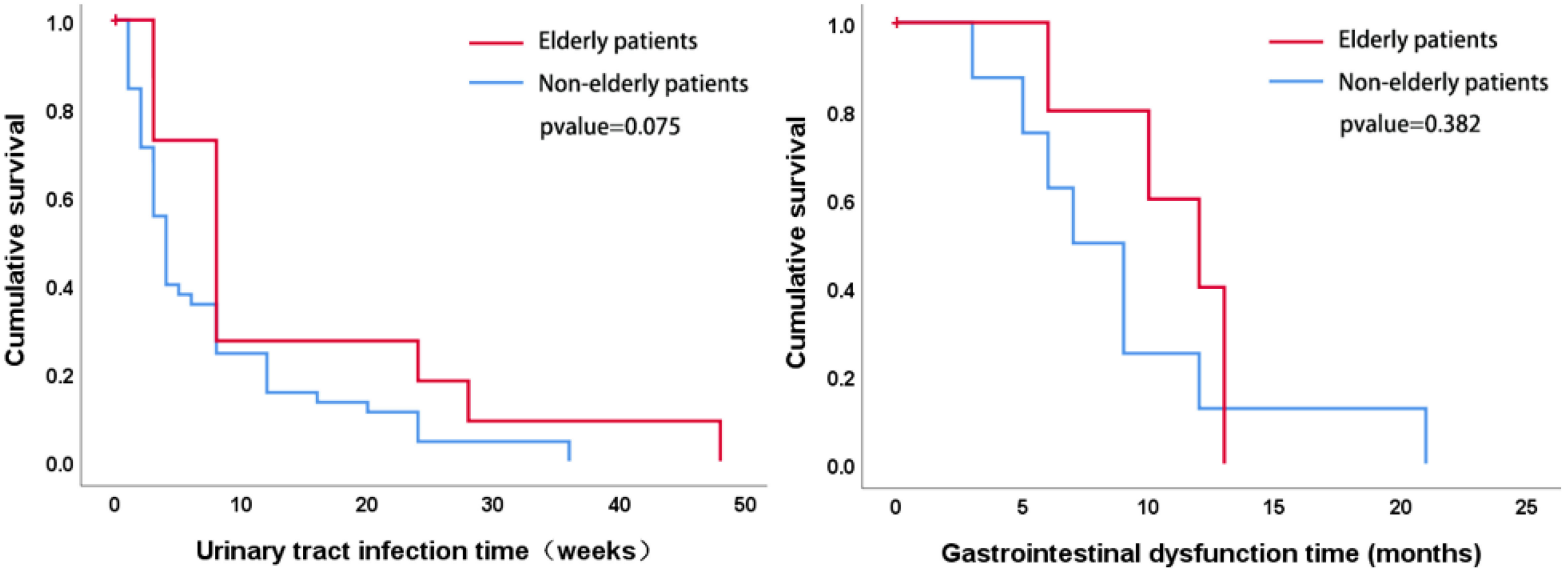
Survival analyses were performed on indicators that included time to first postoperative presentation and survival curves were plotted, which showed no statistically significant differences between the urinary tract infection and bowel obstruction groups.

Comparative analyses of all postoperative complications were conducted between groups, including urinary tract infections, gastrointestinal dysfunction, and stoma-related issues observed during the final week of follow-up. A modified Clavien classification system was used to evaluate these complications, as detailed in **Table 4**. Statistically significant differences were noted in the incidence of stoma inflammation and in the severity according to the modified Clavien classification. Older patients exhibited a lower incidence of stoma inflammation (15% vs. 32%; p=0.04) and higher complication severity, as indicated by the Clavien classification scores at 30 days (p=0.001) and 90 days (p=0.001) compared to the non-elderly group. However, there were no statistically significant differences in the rates of urinary tract infections (15% vs. 22%; p=0.398), gastrointestinal dysfunction (9% vs. 3%; p=0.165), stoma obstruction (3% vs. 4%; p=0.734), and stoma bleeding (12% vs. 24%; p=0.125) at follow-up. An analysis of the factors influencing stoma inflammation is shown in **Supplementary Table 3**, which shows that a higher percentage of stoma discs were replaced by others in the elderly group than in the non-elderly group (patients replaced themselves [9% vs 60%], cooperatively [36% vs 40%], and by others [55% vs 0%]; P<0.001), with the same frequency of replacement (less than 1 week [52% vs 37%], 1-2 weeks [37% vs 55%], greater than 2 weeks [21% vs 8%]; P= 0.968). Defining the emergence of the first postoperative urinary tract infection and gastrointestinal dysfunction as the outcome, and the time of the first occurrence of both complications as the time of the occurrence of the outcome, the survival analysis of postoperative urinary tract infection and postoperative gastrointestinal dysfunction was carried out and the survival curves were plotted, and the results showed that there was no statistically significant difference between the groups of urinary tract infection and intestinal obstruction, as shown in **Figure 1**.

**Table 4:** Surgical safety - postoperative complications and scoring.

		Entire cohort (n=152)	<70 (n=119)	≥70 (n=33)	Pvalue
Urinary tract infection		59 (39)	48 (40)	11 (33)	0.465
gastrointestinal dysfunction		27 (18)	21 (18)	6 (18)	0.943
stoma problems		53 (35)	45 (38)	8 (24)	0.101
	inflammation	43 (28)	38 (32)	5 (15)	0.04
	obstruction	6 (4)	5 (4)	1 (3)	0.734
	bleeding	32 (21)	28 (24)	4 (12)	0.125
30days CCS					0.174
	0	82 (54)	66 (55)	16 (48)	
	1	19 (13)	16 (13)	3 (9)	
	2	33 (22)	29 (24)	4 (12)	
	3	1 (1)	1 (1)	0	
	4	16 (11)	7 (6)	9 (27)	
30days Grade					0.001
	Low grade (CCS<3)	135 (89)	111 (93)	24 (73)	
	High grade (CCS≥3)	17 (11)	8 (7)	9 (27)	
90days CCS					0.317
	0	75 (49)	59 (50)	16 (48)	
	1	17 (11)	15 (13)	2 (6)	
	2	42 (28)	37 (31)	5 (15)	
	3	1 (1)	1 (1)	0	
	4	16 (11)	7 (6)	9 (27)	
90days Grade					0.001
	Low grade (CCS<3)	135 (89)	111 (93)	24 (73)	
	High grade (CCS≥3)	17 (11)	8 (7)	9 (27)	

Counts are expressed as number (percentage).

### 3.3 Surgical outcomes

**Figure 2:**
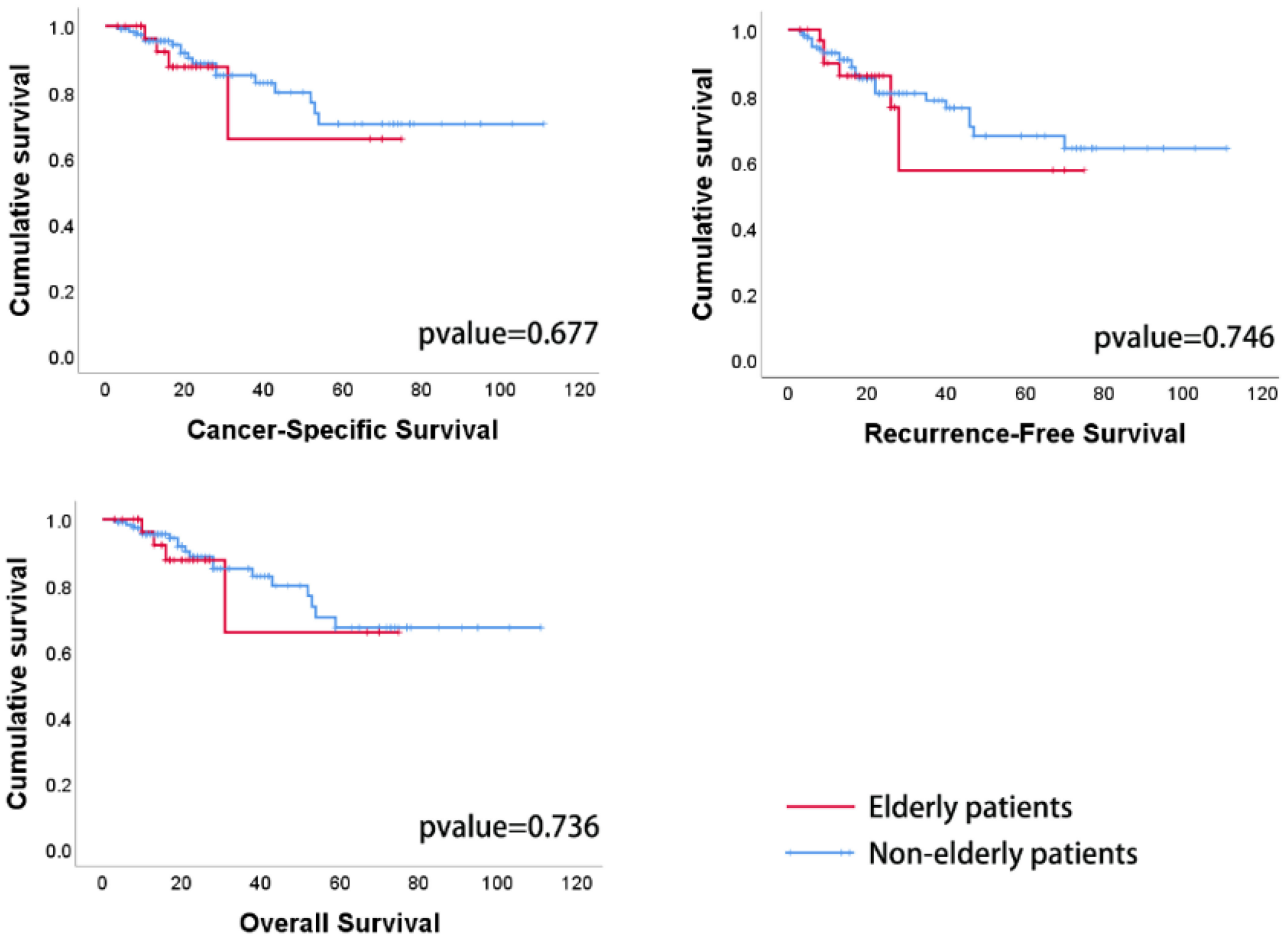
Patient survival times are expressed in months. There were no statistically significant differences in CSS, RFS, or OS between elderly and nonelderly patients.

The analysis of the treatment effect primarily focused on the surgical outcomes, clinical survival, and patients’ FACT-BL scores. Surgical outcomes compared included the number of cleared lymph nodes, the percentage of pathologically positive lymph nodes, 1-year and 3-year postoperative survival rates, rates of urethral recurrence, and distant metastasis. The between-group analysis showed no statistically significant differences in the number of cleared lymph nodes (14.06 vs. 12.19; p=0.44), percentage of positive lymph nodes (4.7% vs. 3.7%; p=0.937), 1-year (96.9% vs. 95.8%; p=0.76) and 3-year survival rates (87.9% vs. 89.9%; p=0.74), urethral recurrence (0% vs. 4.38%; p=0.23), and distant metastasis rate (15.15% vs. 15.96%; p=0.91), as presented in **Table 5**. Additionally, survival analyses comparing cancer-specific survival (CSS), recurrence-free survival (RFS), and overall survival (OS) over an average follow-up of 34 months (**Figure 2**) indicated no significant differences in therapeutic efficacy between elderly and non-elderly patients. Furthermore, comparisons of FACT-BL scores revealed mean total scores of 111 for non-elderly patients and 112 for elderly patients, with no significant differences across the dimensions of physical, social/family, emotional, functional health, and specific concerns about bladder cancer, as detailed in **Table 5**.

**Table 5:** Surgical efficacy.

		Entire cohort (n=152)	<70	≥70	Pvalue
lymphatic node					
	Lymph nodes	12.6	12.19	14.06	0.44
	Proportion of metastatic lymph nodes (%)	3.9	3.7	4.7	0.937
1year survival rate (%)		96	95.8	96.9	0.76
3year survival rate (%)		89.5	89.9	87.9	0.74
Urethral recurrence rate (%)		3.28	4.38	0	0.23
Distant metastasis rate (%)		15.78	15.96	15.15	0.91
FACT-BL					
	physical health	25 (22-26)	25 (22-26)	25 (20.5-26.5)	0.948
	social/family health	21 (19-22)	21 (19-22)	21 (18.5-22)	0.501
	emotional health	21 (18-22)	21 (18-22.5)	20 (18.5-21.5)	0.294
	functional health	22 (20-25)	22 (20-24)	23 (21-25)	0.257
	bladder cancer-specific concerns 1guanguanzhuzhu 注	27 (25-28.25)	27 (25-29)	27 (25.5-28)	0.855
	overall score	116 (108.2-120)	116 (107.5-120.5)	117 (107-120)	0.777

Lymph node counts are expressed as mean values, percentage of positive lymph nodes, 1-year postoperative survival, 3-year postoperative survival, urothelial metastasis, and distant metastasis as percentages; FACT-Bl scores are expressed as median (interquartile spacing).

### 3.4 External validation of efficacy

**Figure 3:**
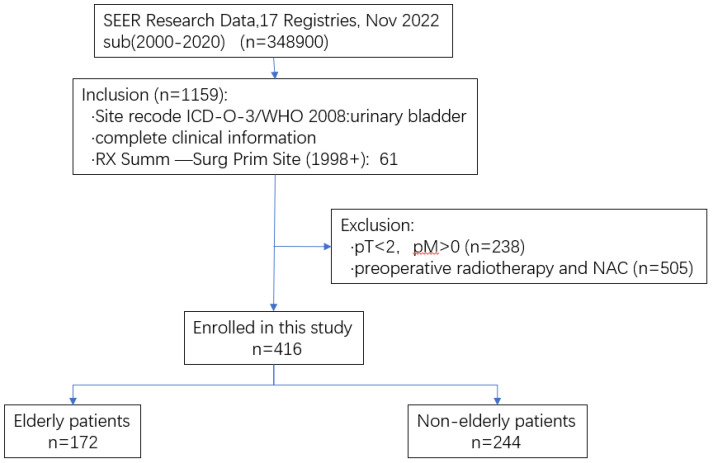
Flow diagram of selecting patients.

**Figure 4:**
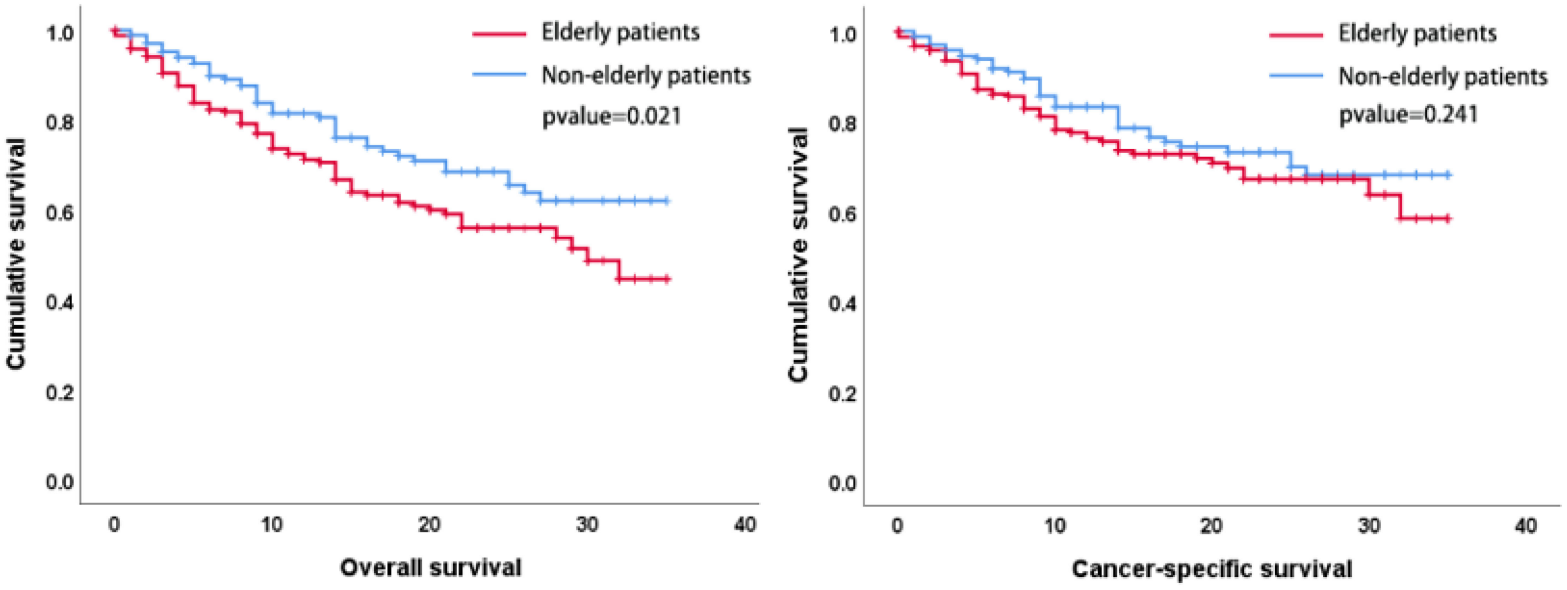
Patient survival times are expressed in months. The OS was shorter in elderly patients (p=0.021) than in non-elderly patients, but the difference in CSS was not statistically significant (p=0.241).

A total of 416 patients were included in the Seer database, including 172 patients (41%) in the non-elderly group and 244 patients (59%) in the elderly group, and the process of screening and grouping of patients is shown in **Figure 3**. There was no statistically significant difference in the gender composition, T-stage, and N-stage between the two groups of patients, and the results are shown in **Table 6**. The Kaplan-Meier method was used to compare the OS and CSS of elderly patients with those of non-elderly patients, and the OS was shorter in elderly patients (p= 0.021) than in non-elderly patients, but the difference in CSS was not statistically significant (p=0.241), and the surgical efficacy of the elderly group was comparable to that of the non-elderly group, and the externally validated results were basically in line with the results of the analysis of our center (**Figure 4**).

**Table 6:** Clinical and pathological characteristics of patients in the SEER database.

		Entire cohort (n=416) (n=152)	<70(n=172)	≥70(n=244)	Pvalue
Male		284 (68)	111 (64)	173 (71)	0.169
Pathologic T stage					0.336
	Tis	9 (2)	1 (0.6)	8 (3)	
	T2	169 (41)	70 (40.7)	99 (41)	
	T3	171 (41)	70 (40.7)	101 (41)	
	T4	67 (16)	31 (18)	36 (15)	
Pathologic N stage					0.965
	N0	308 (74)	128 (74)	180 (74)	
	N1	45 (11)	18 (11)	27 (11)	
	N2	44 (10)	16 (9)	28 (11)	
	N3	17 (4)	8 (5)	9 (4)	
	Nx	2 (1)	2 (1)	0	

Counts are expressed as number (percentage).

## 4 Discussion

RC is the current standard procedure for the treatment of MIBC, especially with the current development of minimally invasive surgical techniques in urology, more and more relevant studies have reported the advantages of LRC and RARC over traditional ORC in terms of reducing intraoperative bleeding, perioperative blood transfusion, and many other aspects (13–15). The minimally invasive surgical approach to RC seems to have become a paradigm for doctors and patients in clinical practice, but there has been no uniform standard on how to divert urinary flow after RC, whether minimally invasive or open surgery. The mode of urinary diversion has been associated with a variety of postoperative complications, and the selection of an appropriate urinary diversion for a patient requires careful consideration of a number of factors, including the patient’s age, concomitant diseases, preoperative renal function, and life expectancy.CU, IC, and *in situ* neobladder are the common choices for urinary diversion in current clinical practice. Although IC has the disadvantages of longer operative time, higher bleeding, and longer recovery of bowel function than CU, and does not have the advantages of *in situ* neobladder in controlling urination and eliminating the need for urinary bags, IC is still the first choice for urinary diversion in elderly patients due to the fact that it does not require frequent catheter changes, long-term functional exercise, relatively fewer complications, and easier care. For the assessment factor of age, there are now many studies comparing cross-sectional efficacy and safety between different surgical procedures in elderly patients with MIBC (16–20). However, there is still a gap in studies comparing the differences of a single surgical procedure under different age subgroups. Therefore, a longitudinal analysis of the efficacy and safety of RC with IC in the treatment of different age subgroups to determine whether old age should be used as an exclusion criterion for the selection of IC is of great clinical significance and reference value.

To define the grouping criteria of elderly and non-elderly patients, we conducted a review of the literature on RC. The global median age for bladder cancer patients is 73 years, while the average and median ages of the 349,800 patients in the SEER database are 69 years and 70 years, respectively. Based on these statistics and current published research, we determined that using 70 years as the cut-off point for classifying elderly patients is a more appropriate choice. Although neoadjuvant chemotherapy (NAC) has been confirmed by several studies for its role in MIBC prognosis (21–23), NAC still suffers from low adherence among patients and age factors can influence patients’ choice of NAC (24), so the criteria were set to exclude patients who had undergone preoperative NAC to remove the effect of NAC on the survival of patients in the two groups, and to compare only the therapeutic impact of RC with IC in patients of different age subgroups.

According to this study’s analysis, both our center’s data and external validation indicate that the difference in treatment efficacy across different age subgroups is not statistically significant, demonstrating that elderly patients exhibit similar efficacy to non-elderly patients. In terms of surgical safety, our center identified three notable differences between the two age groups: variations in CCS grading, the need for postoperative ICU transfer, and the occurrence of stoma inflammation during the last week of follow-up.

We analyzed the reasons behind the higher CCS grading observed in elderly patients and found that this was attributable to a greater proportion of elderly patients receiving a score of 4. This higher scoring was linked to a higher rate of ICU transfers among the elderly, which coincides with the factors discussed in the ICU transfer analysis. These interconnected issues are discussed in further detail in the following section. In the study by Kai W Cheng et al, advanced age was identified as an influencing factor for unplanned postoperative transfer of patients to the ICU (25), whereas Wei-Yu Lin demonstrated in his study that advanced age does not lead to prolonged ICU stays (26). The results of their study are generally consistent with the results of the analysis of our center’s data. For the comparison of the risk of transfer to the ICU, there were no statistically significant differences between groups in terms of procedure, operator, and operator proficiency, and there were no significant confounders or biases in the analyses for whether or not to transfer to the ICU. Subsequently, we investigated the reasons for transferring patients to the ICU, and among the patients transferred to the ICU due to postoperative anesthesia factors that could not maintain vital signs accounted for 71% of all patients, followed by postoperative delirium (18%). All patients transferred to ICU could be transferred back to the normal ward within 2 days on average, also there were no patients in the study who had injuries or prolonged hospital stay due to transfer to ICU, and with the combined score we concluded that the risk of postoperative transfer to ICU in elderly patients was higher but it did not affect the overall surgical safety of elderly patients.

Currently, few studies have been conducted on irritant contact dermatitis of the ileal passage and surrounding skin after IC. Thirty-three percent of the patients in our center’s follow-up results had experienced peristoma itching or developed peristoma papules and herpes in the last 1 week. Grouped according to age, the analyses showed that the percentage of older patients presenting with contact dermatitis in the last week was even lower. In response to this counterintuitive finding, we further analyzed the factors that may have contributed to the offset, including differences in stoma replacement patterns and frequency of replacement in different age groups. The results showed that a higher proportion of older patients had their stomas changed by a chaperone or with the assistance of a chaperone and that more careful inspection of the stoma status and more careful cleaning may have contributed to the lower incidence of irritant dermatitis in the older group. The lack of recall of this symptom due to reduced skin sensation in the elderly group cannot be excluded.

In summary, we concluded that although there were differences in the occurrence of postoperative complications in the elderly group of patients compared with the non-elderly group, the associated health risks due to complications were still within manageable limits. Combining the survival status of the elderly group with that of the non-elderly group, we conclude that the efficacy and safety of RC with IC for the treatment of MIBC in the elderly is consistent with that of the non-elderly patients.

Finally, there are some limitations of our study. Firstly, due to the small sample size, we were unable to stratify the patients into more detailed age groups to investigate the differences in safety and efficacy between different age groups. Second, clinical retrospective studies may be affected by confounding factors, and the safety of surgery for elderly patients needs to be further verified in multicenter prospective studies.

## 5 Conclusion

For longitudinal age subgroups, there was concordance in the efficacy of RC with IC for the treatment of MIBC in the elderly versus the non-elderly. Although there are surgical risks in elderly patients, they do not affect the overall recovery of patients, and the difference in safety between elderly and nonelderly patients is not significant. Based on the results of the current study, old age should not be used as an absolute exclusion criterion for RC with IC.

## Data availability statement

The original contributions presented in the study are included in the article/supplementary material. Further inquiries can be directed to the corresponding author/s.

## Ethics statement

The studies involving humans were approved by The Ethics Committee of the Cancer Hospital of Harbin Medical University. The studies were conducted in accordance with the local legislation and institutional requirements. The participants provided their written informed consent to participate in this study.

## Author contributions

HZ: Data curation, Methodology, Writing – original draft, Writing – review & editing. AL: Writing – review & editing. WW: Investigation, Writing – review & editing. SX: Data curation, Software, Writing – review & editing. CL: Writing – review & editing. LT: Writing – review & editing.
