# Supplementary material for: Efficacy and safety of radical cystectomy with ileal conduit for muscle-invasive bladder cancer in the elderly: a multicenter retrospective study
Source: Front Oncol. 2024 Jul 23;14:1402360. doi: 10.3389/fonc.2024.1402360 (PMC11300965; doi:10.3389/fonc.2024.1402360)
Supplement: Supplementary file 1 [file Table_1.pdf]

sTable 1 Impact of surgical approach and operator on transfer to the ICU

		Not transferred to ICU	transferred to ICU	P value
Surgical approach				0.298
	Open	49	4	
	Laparoscopy	86	13	
Operator				0.44
	1	1	0	
	2	13	1	
	3	27	1	
	4	94	15	

sTable 2 Reasons for transfer to ICU and length of stay in ICU

Age cohort	Patient ID	Age	Reason	Length of in ICU stay (days)
Non-elderly patients	1	69	Delayed awakening from anesthesia	1
	2	61	Delayed awakening from anesthesia	2
	3	67	Delayed awakening from anesthesia	1
	4	58	Delayed awakening from anesthesia	5
	5	67	Delayed awakening from anesthesia	1
	6	64	Delayed awakening from anesthesia	1
	7	62	Delayed awakening from anesthesia	1
Elderly patients	1	78	Postoperative delirium	1
	2	71	Postoperative delirium	1
	3	85	Postoperative delirium	1
	4	77	Delayed awakening from anesthesia	1
	5	75	Delayed awakening from anesthesia	1
	6	75	Post-operative bleeding	5
	7	70	Heart failure	2
	8	77	Delayed awakening from anesthesia	1
	9	78	Delayed awakening from anesthesia	1
	10	72	Delayed awakening from anesthesia	1

sTable 3 Frequency and pattern of changing ileal conduit storage bags in patients

		Entire cohort	<70(n=119)	≥70(n=33)	Pvalue
Frequency					0.685
	<1 weeks	62(41)	45(37)	17(52)	
	1-2 weeks	74(48)	65(55)	9(37)	
	>2 weeks	16(11)	9(8)	7(21)	
Pattern					<0.001
	oneself	74(48)	71(60)	3(9)	
	cooperation	60(39)	48(40)	12(36)	
	others	18(12)	0	18(55)	
